# Comparing Gut Microbiome in Mothers’ Own Breast Milk- and Formula-Fed Moderate-Late Preterm Infants

**DOI:** 10.3389/fmicb.2020.00891

**Published:** 2020-05-26

**Authors:** Ziyi Wang, Achal Neupane, Richard Vo, Jessica White, Xiuqing Wang, Shin-Yi Lee Marzano

**Affiliations:** ^1^Department of Biology and Microbiology, South Dakota State University, Brookings, SD, United States; ^2^Department of Pediatrics, Sanford Children’s Hospital, Sanford USD Medical Center, Sioux Falls, SD, United States; ^3^Sanford School of Medicine, University of South Dakota, Vermillion, SD, United States; ^4^Department of Agronomy, Horticulture, and Plant Science, South Dakota State University, Brookings, SD, United States

**Keywords:** infant gut microbiome, metatranscritome, breastfeeding, moderate-late preterm infants, 16S rRNA gene

## Abstract

Gut microbiome plays an important role in adult human health and diseases. However, how nutritional factors shape the initial colonization of gut bacteria in infants, especially in preterm infants, is still not completely known. In this study, we compared the effects of feeding with mothers’ own breast milk (MBM) and formula on the initial composition and gene expression of gut bacteria in moderate–late preterm infants. Fecal samples were collected from ten formula-fed and ten MBM healthy infants born between 32 and 37 weeks’ gestation after they reached full-volume enteral feedings. Total DNAs were extracted from fecal samples for amplicon sequencing of 16S ribosomal RNA (rRNA) gene and total RNA with rRNA depletion for metatranscriptome RNA-Seq 16S rRNA gene amplicon sequencing results showed that the alpha-diversity was similar between the MBM- and formula-fed preterm infants, but the beta-diversity showed a significant difference in composition (*p* = 0.002). The most abundant taxa were *Veillonella* (18.4%) and *Escherichia*/*Shigella* (15.2%) in MBM infants, whereas the most abundant taxa of formula-fed infants were *Streptococcus* (18.6%) and *Klebsiella* (17.4%). The genera *Propionibacterium*, *Streptococcus*, and *Finegoldia* and order *Clostridiales* had significantly higher relative abundance in the MBM group than the formula group, whereas bacteria under family *Enterobacteriaceae*, genera *Enterococcus* and *Veillonella*, and class *Bacilli* were more abundant in the formula group. In general, microbiomes from both diet groups exhibited high functional levels of catalytic activity and metabolic processing when analyzed for gene ontology using a comparative metatranscriptome approach. Statistically, the microbial genes in the MBM group had an upregulation in expression related to glycine reductase, periplasmic acid stress response in Enterobacteria, acid resistance mechanisms, and L-fucose utilization. In contrast, the formula-fed group had upregulations in genes associated with methionine and valine degradation functions. Our data suggest that the nutritional source plays a role in shaping the moderate–late preterm gut microbiome as evidenced by the differences in bacterial composition and gene expression profiles in the fecal samples. The MBM group enriched *Propionibacterium*. Glycine reductase was highly upregulated in the microbiota from MBM along with the upregulated acid stress tolerance genes, suggesting that the intensity of fermentation process was enhanced.

## Introduction

The gut microbiome has been increasingly found to affect human health. Although individual differences exist, core microbiome including *Firmicutes* and *Bacteroidetes* are considered crucial to the maintenance of health in adults (Eckburg et al., 2005; Turnbaugh and Gordon., 2009; Tap et al., 2009; Qin et al., 2010). Compared to the gut microbiomes of adults, infants, especially preterm neonates, have relatively dynamic and unstable microbiomes. Multiple factors, including genetic factors (Stewart et al., 2005) and environmental variables such as nutrition source, antibiotic use, and delivery method, could potentially affect infants’ microbiome composition and richness (Palmer et al., 2007; D’Argenio and Salvatore, 2015; Lim et al., 2016). Transient compositional changes in the intestinal microbiome may impact the neonate differently than the adult as the neonatal gut is not yet fully developed (Voreades et al., 2014; Gritz and Bhandari, 2015). During infancy, especially in preterm neonates, the primary source of enteral nutrition is breast milk and/or formula. Formula is the preferred alternative when mothers cannot produce enough breast milk, which is a common challenge to mothers of preterm infants (Radtke, 2011). Formula is considered a balanced nutrient source and may promote higher growth rates of preterm infants than breast milk alone (Gross, 1983; Quigley and McGuire, 2014). In recent years, however, our understanding of the complex composition of breast milk and the mother–infant dyad has led to an increased focus on the benefits of breastfeeding (Hanson et al., 1978; Peterson et al., 1998; Hamosh, 2001; Oddy, 2002; Wold and Adlerberth, 2002). Studies demonstrate a role of breast milk in promoting health in infants, which includes lowering the risk of necrotizing enterocolitis (NEC) (Lucas and Cole, 1990; Boyd et al., 2007; Abrams et al., 2014; Quigley and McGuire, 2014), type I diabetes (Virtanen et al., 1992), and Crohn’s disease (Bergstrand and Hellers, 1983) and promoting neurodevelopment (Lucas et al., 1992) as well as reducing the rate of obesity in childhood (Armstrong et al., 2002).

The mechanisms by which breast milk contributes to human health are still unclear. Studies indicate that both extremely low birth weight preterm infants and very preterm infants fed with breast milk exhibit increased gut microbial alpha-diversity compared to formula-fed counterparts of 20–30 days (Gewolb et al., 1999; Cong et al., 2016). *Bifidobacteria*, which are probiotic in the human gut (Collins and Gibson, 1999; O’Callaghan and van Sinderen, 2016), can colonize the gut of breast milk-fed full-term newborns easier than the gut of formula-fed infants (Bullen et al., 1976; Stark and Lee, 1982; Mevissen-Verhage et al., 1987; Balmer and Wharton, 1989; Harmsen et al., 2000; Bezirtzoglou et al., 2011; Lee et al., 2015). Specifically, *Bifidobacterium longum* was more abundant in the gut of breast milk-fed full-term infants than that of formula-fed infants without probiotic supplements (Lee et al., 2015), and *Bifidobacterium* spp. decreased after changing the nutrition from breast milk to formula milk (Davis et al., 2016). Another genus, *Lactobacillus* (Collins and Gibson, 1999), at around 3% of the microbiome population in breast milk (Urbaniak et al., 2016), was more abundant in breast milk-fed full-term infants’ than in formula-fed infants’ fecal samples (Madan et al., 2016) and also decreased after changing the nutrition source from breast milk to formula (Davis et al., 2016). On the contrary, potentially pathogenic genera, such as *Clostridia*, *Klebsiella*, *Veillonella*, and *Bacteroides*, colonize better in formula-fed full-term infants’ gut than that of breast milk-fed infants (Benno et al., 1984; Mackie et al., 1999; Boyd et al., 2007; Adlerberth and Wold, 2009; Bezirtzoglou et al., 2011; Lee et al., 2015). The difference noted between the two groups has led to an interest in manipulating the infant microbiome through the use of probiotics.

Recently, microbiome analyses to predict microbial function in the gut have been highlighted. Probiotic metabolites can promote an anti-inflammatory function by producing short-chain fatty acids (Fu et al., 2017; Xiong et al., 2017). Predictive functional pathway analyses of microbiome based on 16S rRNA gene amplicon sequencing inferred that exclusively breast milk-fed infants had higher relative abundances of microbial membrane transport and lower energy metabolism than formula-fed infants (Thompson et al., 2015). However, a meta-analysis study inferred that non-exclusively breast milk-fed infants had more relative abundances of microbial functions of carbohydrate metabolism but reduced lipid metabolism, vitamin metabolism, and detoxification than exclusively breast milk-fed infants (Ho et al., 2018). Metatranscriptomics provides clearer pictures of microbiome functionality than predicted functions from 16S rRNA gene amplicon sequencing (Lavelle and Sokol, 2018). By metatranscriptome analysis, functional human gut microbiota can be directly described without functional inference based on the phylogeny (Booijink et al., 2010; Gosalbes et al., 2011; Franzosa et al., 2014; Hugenholtz et al., 2017).

Many previous studies have examined the microbiome in the first month or the first year of full-term infants (Balmer and Wharton, 1989; Stark and Lee, 1982; Penders et al., 2006; Li et al., 2014; Lim et al., 2015, 2016; McCann et al., 2018; Pannaraj et al., 2018). Yet, 10% of the United States population in 2018 was born preterm, with the majority of preterm infants born moderate–late preterm (Hamilton et al., 2019). To our knowledge, however, only several studies have focused on this group (Forsgren et al., 2017; Chernikova et al., 2018; Zwittink et al., 2018). Specifically, an investigation on the healthy population of this group will lay the groundwork for the study of preterm infant dysbiosis-related diseases using a dual-cohort approach to better tease out nutrition effects (Furse et al., 2018). Therefore, the goal of the present work was to compare the composition and function of gut microbiome as related to the nutritional source, i.e., mothers’ own breast milk (MBM) versus formula, in moderate–late preterm neonates. The approach we have used in this study includes the high-throughput sequencing of 16S rRNA gene amplicon sequencing and comparative metatranscriptomics. We aimed to determine potentially probiotic taxa in the MBM group and to unveil the functional differences in moderate–late preterm infant gut microbiome between the two nutrition sources.

## Materials and Methods

### Sample Collection

The Sanford Health IRB, ID STUDY00000829IRA, approved all the experiments that involved human subjects in this study. Twenty otherwise healthy infants without congenital anomalies who were delivered moderate–late preterm (32 0/7 to 36 6/7 weeks’ gestation) were recruited to the study. Fecal specimens were collected after infants reached full-volume enteral feedings. Following parental consent, all enrolled infants were assigned an ID number. The descriptions and summary of infants are shown in Table 1 and Supplementary Table S1. Ten of the twenty infants were MBM (gestational age: 33.7 ± 1.26 weeks), and ten were formula-fed (gestational age: 34 ± 1.5 weeks). All infants enrolled to the breast milk arm of the study received their own mother’s milk. None of the infants enrolled to the MBM group received a formula feeding prior to sample collection; however, these infants did receive breast-milk feedings fortified with either human milk fortifier or Neosure infant formula as per unit standard practice. Infants enrolled to the formula-fed group did not receive any breast milk prior to sample collection. All mothers of enrolled infants received antibiotics prior to delivery for either concern for infection or as prophylaxis before cesarean section. Eleven of twenty infants received Ampicillin/Gentamicin antibiotics while undergoing observation and evaluation for neonatal sepsis. None of these infants had a positive blood culture. Six of twenty infants were delivered vaginally, and fourteen were born via Cesarean section (C-section). Six recruited infants were Native American, and fourteen infants were Caucasian, with five pairs of twins among them: infants #7 and #8; #9 and #10; #18 and #19; #21 and #22; and #23 and #24. For each infant, stool samples were collected at two time points at a minimum of 24 h apart. The first samples were collected at an average of 15 days after birth, and the second collection of samples occurred at an average of 17 days after birth. Each collected sample was stored in DNA/RNA shield tubes (Zymo Research), which contain a DNA/RNA shield to reduce nucleic acid degradation. All samples were stored at 4°C.

**TABLE 1:** Demographic and clinical information of infants enrolled in the study.

Infants ID (#)	Nutrition	Gender	Infant antibiotic use	Delivery type	Race/Ethnicity	Twin pair
1	Formula	F	0	Cs	C	
2	Breast milk	M	1	Cs	C	
3	Breast milk	F	0	Cs	C	
7	Formula	F	0	V	N	A
8	Formula	M	0	V	N	A
9	Breast milk	M	0	Cs	C	B
10	Breast milk	M	0	Cs	C	B
11	Breast milk	F	1	V	C	
13	Breast milk	F	0	Cs	C	
15	Formula	M	0	V	N	
16	Breast milk	F	1	V	C	
18	Formula	M	1	Cs	N	C
19	Formula	F	1	Cs	N	C
20	Breast milk	F	1	V	C	
21	Breast milk	F	1	Cs	C	D
22	Breast milk	F	1	Cs	C	D
23	Formula	M	1	Cs	C	E
24	Formula	M	1	Cs	C	E
25	Formula	F	1	Cs	N	
27	Formula	M	0	Cs	C	

*Gender: F, female; M, male. Infant antibiotic use: 0, non-antibiotics; 1, received ampicillin/gentamicin antibiotic treatment. Delivery type: Cs, cesarean section; V, vaginal delivery. Race/Ethnicity: C, Caucasian; N, Native American. Twins: five pairs of twins were included – A, B, C, D, and E.*

### Statistical Analysis

GraphPad Prism software was used to perform unequal variance (Welch) unpaired *t*-tests to examine the statistical significance of gestational age, birth weight, and the first and second sample collection times between the two groups of enrolled infants.

### DNA/RNA Extraction

Total DNA and RNA were simultaneously extracted from fecal samples stored in DNA/RNA Shield tubes supplied in the DNA/RNA Microbiome Mini Kit (ZRC188678, ZymoBIOMICS^TM^) following the manufacturer’s instruction. The concentrations of the extracted DNA/RNA were quantified with both Qubit 3.0 Fluorometer (Invitrogen^TM^) and NanoDrop^TM^ Spectrophotometer (Thermo Scientific^TM^).

### 16S rRNA Gene Amplicon Sequencing and Bioinformatics Analyses

The 16S rRNA gene amplicon sequencing of the V3–V4 segments was performed as described previously using MiSeq Next Generation Sequencer at the University of Minnesota Genomics Center (Gohl et al., 2016).

An average of 44,558 paired-end reads per sample were obtained and uploaded to Sequence Read Archive (SRA) of the National Center for Biotechnology Information (NCBI) under accession PRJNA515307. Sequences were filtered at Phred 33 and demultiplexed by Quantitative Insights Into Microbial Ecology 2 (QIIME2) (Bolyen et al., 2018). The amplicon sequence variants (ASVs) were obtained following the QIIME2 tutorial against the NCBI nt database, and taxonomic analysis was performed using GreenGenes (version 13.8) (McDonald et al., 2012). Alpha- and beta-diversity analysis, PERMANOVA analysis, ANCOM (Mandal et al., 2015), and differential abundance analysis with gneiss were also performed through QIIME2. The LEfSe algorithm (Segata et al., 2011) was used after QIIME2 with default parameters to determine the differences in biomarkers. Additionally, 16S raw data were also analyzed using USEARCH (Edgar, 2013). Briefly, sequencing data were trimmed and filtered with expected errs <1 by USEARCH. Ninety-seven percent of the operational taxonomic unit (OTU) table, alpha- and beta-diversity, and taxonomy with Ribosomal Database Project (RDP) training set v16 (rdp_16s_v16.fa) database (Cole et al., 2009) were performed by using the UPARSE pipeline of USEARCH.

### RNA-Seq and Bioinformatics Analyses

Human rRNAs were depleted from total RNA using rRNA Depletion Kit (Human/Mouse/Rat) (E6310L, NEBNext^®^) following the manufacturer’s instructions. Library preparation was performed by using the Directional RNA Library Prep Kit for Illumina kit (E7760S, NEBNext^®^). HiSeq 4000 (per lane 100 bp paired-end) was performed at the Roy J. Carver Biotechnology Center at the University of Illinois at Urbana-Champaign. The total RNA from 16 of the 20 infants was sequenced in two runs of RNA-seq. In the first run, samples from #1, #2, #3, #7, #8, #9, #10, #11, and #13 infants included two time points each. In the second run of RNA-seq, samples from #18, #19, #20, #21, #22, #23, and #24 infants were collected with two time points pooled together after we determined that the two time points (8∼12 h apart) had similar microbial compositions in the first run.

The metatranscriptome data were trimmed by Trimmomatic (version 0.39) (Bolger et al., 2014), and then DIAMOND (version 0.9.24) (Buchfink et al., 2015) was used to align DNA reads with the NCBI nr database. The data will be available once accepted. MEGAN6 (Huson et al., 2007) was used to parse the aligned output from DIAMOND to obtain bacterial phylogeny and gene ontology results against InterPro databases. The SAMSA2 pipeline (version 2.2.0) (Westreich et al., 2018) was followed by preprocessing steps as shown in the manual with some minor modifications. Changes included joining the paired-end sequencing libraries with Fastq-Join (version 1.3.1) (Aronesty, 2013), adaptor trimming with Trimmomatic (version 0.39), removing bacteria rRNA with SortMeRNA (version 2.1) (Kopylova et al., 2012) against the SILVA database (version 1.1.9) (Pruesse et al., 2007), mapping by DIAMOND (version 0.9.24) against the NCBI RefSeq database (Tatusova et al., 2013), and mapping to the SEED Subsystems database (Overbeek et al., 2013). The versions of RefSeq and SEED Subsystems databases were obtained via BioShare (“Databases Used” in README.md)^1^ and downloaded by running the “full_database_download.bash” script as part of SAMSA2. The DIAMOND output files were aggregated by using R and Python scripts for statistical analysis and DESeq2 differential expression as part of the SAMSA2 pipeline.

## Results

### Demographic Information of Enrolled Infants

Twenty infants were recruited to this study. Ten infants were MBM fed, and ten infants were formula-fed. Informed consent was obtained from all participants’ parents. Infant characteristics are summarized in Table 1. An unequal variance (Welch) unpaired *t*-test was used to analyze the differences in demographic information between the two groups. There were no significant differences in gestational age, birth weight, or first and second sampling ages observed between the two groups (Supplementary Table S1). This excludes these potential confounding factors between the treatment groups that may affect the interpretation of the results.

### Effect of Nutrition Source on the Alpha- and Beta-Diversity of Gut Bacterial Composition

**FIGURE 1:**
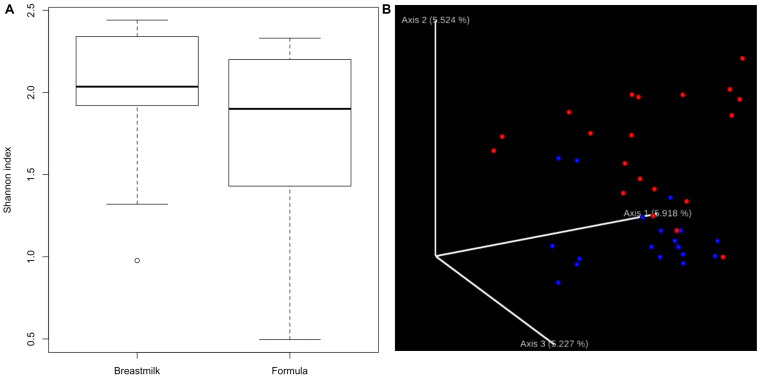
Alpha- and beta-diversity. **(A)** Boxplots showing the bacterial alpha-diversity between the two diet groups. There is no significant difference in Shannon index of alpha-diversity between MBM and formula groups. **(B)** Jaccord plot of beta-diversity presented by EMPeror. Distinct clustering of beta-diversity was observed between MBM (red) and formula groups (blue).

We first compared the alpha-diversity of the gut bacterial composition between MBM- and formula-fed infants through both QIIME2 and UPARSE pipeline with 16S rRNA sequencing data. No significant difference in bacterial alpha-diversity between the two diet groups was found (Figure 1A and Supplementary Table S2). There was a relatively large variation among the individuals in the formula-fed group compared to the MBM group.

In addition to alpha-diversity, beta-diversity is another important indicator to determine whether different nutrition sources could have an effect on the microbial composition. As shown in Figure 1B, distinct clusters for each nutrition source were observed. Furthermore, the PERMANOVA analysis of the clustering was significant at *P* < 0.01, supporting that the microbial compositions in MBM and formula groups were significantly different.

### Differences in Fecal Microbiome Composition Between MBM and Formula Groups

**FIGURE 2:**
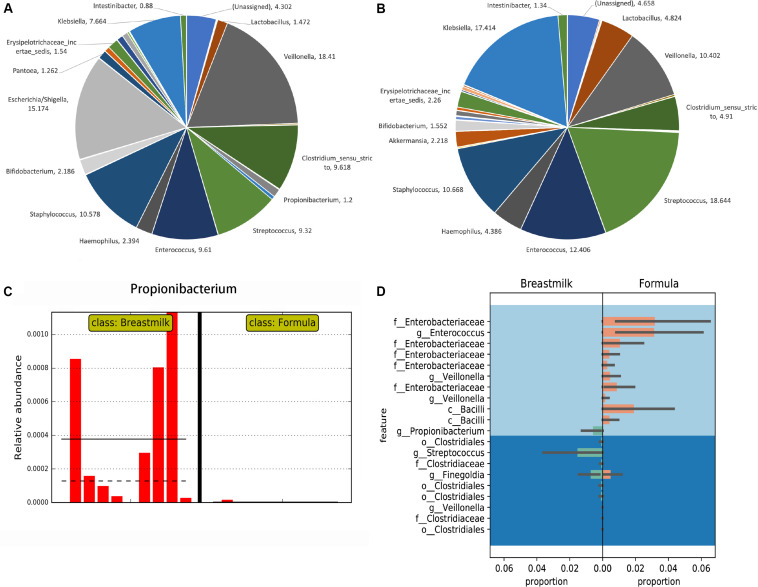
Taxonomy comparisons between MBM- and formula-fed infants based on the 16S rRNA gene amplicon sequencing results. **(A)** Taxonomy pie charts of the proportion in the MBM group in genus level from UPARSE. The number of each sector is the percentage of that bacteria. **(B)** Taxonomy pie charts of the proportion in the formula-fed group in genus level from UPARSE. The number by each section refers to the percentage of that bacteria. **(C)** The LEfSe result on the relative abundance found in *Propionibacterium* between MBM- and formula-fed infants. **(D)** Differential relative abundances of taxa between MBM- and formula-fed infants based on Gneiss.

The OTUs obtained from USEARCH at the phylum level reveal that *Firmicutes* was the most abundant bacterial phylum in the majority of MBM- and formula-fed preterm infants with an average of 67 and 69% of the composition, respectively. *Proteobacteria* was the second most abundant bacteria with a composition of 29 and 25% for the MBM and formula groups, respectively, (Supplementary Figure S1). Syntax taxonomy pie charts from the USEARCH pipeline profiled proportions of different bacteria genus (Figures 2A,B). The *Veillonella* genus and *Escherichia*/*Shigella* genus have the most richness in MBM preterm infants, at 18.41% and 15.17%, respectively. They were followed by *Staphylococcus* (10.58%), *Clostridium* (9.62%), *Enterococcus* (9.61%), and *Streptococcus* (9.32%). In formula-fed preterm infants, *Streptococcus* (18.64%) and *Klebsiella* (17.41%) were the most abundant bacterial genus followed by *Enterococcus* (12.41%), *Staphylococcus* (10.67%), and *Veillonella* (10.4%). *Akkermansia* was unique to the formula-fed group, while *Lactococcus* was unique to the MBM group.

We next performed the LEfSe algorithm for comparative metagenomics analysis to compare the relative abundances between the two groups. The LEfSe results showed that *Propionibacterium* in the MBM group had significantly greater relative abundance than did the formula group (Figure 2C), which was also confirmed by Analysis of Composition of Microbiomes (ANCOM) results from the QIIME2 pipeline. Also, the results from the random forest classifier and otu_select command in the UPARSE pipeline agreed with both the ANCOM and LEfSe analyses that *Propionibacterium* is the most informative OTU that can predict effectively to separate the two groups (Supplementary Tables S3, S4). After using a balanced approach, Gneiss, a longer list of taxa was found to be differentially abundant (Figure 2D). In the formula group, family *Enterobacteriaceae*, genus *Enterococcus*, genus *Veillonella*, and class *Bacilli* were more abundant, whereas order *Clostridiales*, genus *Streptococcus*, and genus *Propionibacterium* were more abundant in the MBM group. LefSe was also used to analyze the differential abundance based on other groupings, i.e., antibiotic, delivery mode, race, and twin factors (Supplementary Figure S2). No significant differences in *Propionibacterium* were found when other groupings were employed.

**FIGURE 3:**
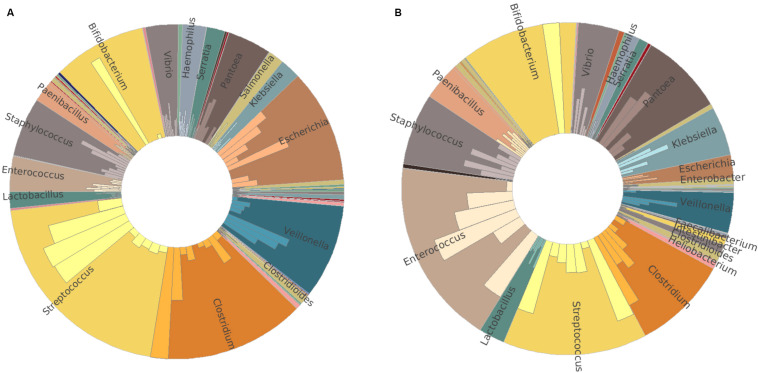
Taxonomy comparison between MBM- and formula-fed infants based on metatranscriptomic results at genus level. **(A)** Relative abundance in the MBM group. **(B)** Relative abundance in the formula-fed group.

To confirm the amplicon sequencing of 16S rRNA gene results, we also performed a metatranscriptomic analysis from 16 of the 20 infants. Similar relative abundance patterns of *Clostridium*, *Streptococcus*, *Veillonella*, *Bifidobacterium*, *Enterococcus*, *Escherichia*, and *Staphylococcus* were obtained by metatranscriptome results in both groups (Figures 3A,B). In addition to these dominators, *Pantoea*, *Vibrio*, and *Paenibacillus* were detected to have more relative abundance profiled by metatranscriptome analysis than those by 16S rRNA gene amplicon sequencing. High individual variations in both bacterial composition and richness were observed (Supplementary Figure S3). *Streptococcus* and *Clostridium* were the most abundant bacterial genera in the MBM group, as well as *Escherichia*, *Veillonella*, and *Bifidobacterium* (Figure 3A). In the formula group, the most abundant bacterial genera were *Enterococcus* and *Streptococcus*, followed by *Bifidobacterium*, *Clostridium*, *Pantoea*, and *Staphylococcus* (Figure 3B).

### Microbial Functions in Infant Stool Samples

The metatranscriptome data was subsequently analyzed through gene ontology analysis by InterPro2GO in MEGAN6. A total of 2,039,141 reads were obtained. 1,647,709 reads were involved in biological processes. 571,096 reads belonged to a cellular component. 1,486,812 reads were associated with molecular function. 225,729 reads were unclassified. Based on the InterPro2GO results, genes related to catalytic activity and metabolic process were over-represented in both MBM-fed and formula-fed infants (Supplementary Figures S4, S5). There were several noticeable differences between the two groups (Supplementary Table S5). The formula group had more intrinsic components of membrane and transport function than did the MBM group. The genes in nucleic acid binding, ion binding, and DNA metabolic process were relatively more prevalent in the MBM group than in the formula group.

To find differential gene expression, the metatranscriptome data was analyzed by SAMSA2. From the heatmap, MBM and formula groups generally clustered separately with few exceptions that the formula group was split into two (Supplementary Figure S6). Based on the DESeq2 result, there were 22 genes differentially expressed (*P*_adj_ < 0.05) with the absolute value of log2 fold change greater than 2. The differentially expressed genes were further categorized into three levels (Table 2) which shows that acid stress and acid resistance mechanisms, as well as L-fucose utilization, increased in the MBM group, whereas valine and methionine degradation increased in the formula group in hierarchy level 2. In hierarchy level 3, the largest upregulation in the MBM group was in the glycine reductase component B gamma subunit (20.327-log2 fold increase), followed by probable glutamate/gamma-aminobutyrate antiporter (9.128-log2 fold increase) and chaperone HdeA (8.802-log2 fold increase). The notable upregulation in the formula group was in methionine gamma-lyase (7.648-log2 fold increase) and ATP-dependent RNA helicase YqfR (2.919-log2 fold increase) compared to the MBM group.

**TABLE 2:** Summary of annotation analysis based on the hierarchy of the SEED Subsystems database showing three levels for each annotated function that have significant difference in expression between breast milk group and formula group (*p*_adj_ < 0.05).

Function names (Level 1)	Function names (Level 2)	Log2 fold change*	Function names (Level 3)	Log2 fold change
Amino acids and derivatives	Glutamine, glutamate, aspartate and asparagine biosynthesis	NA	Glutaminase (EC 3.5.1.2)	4.762
	Methionine degradation	−7.373	Methionine gamma-lyase (EC 4.4.1.11)	−7.648
	Valine degradation	−4.805		
Carbohydrates	L-fucose utilization	6.207	L-fucose isomerase (EC 5.3.1.25)	7.777
			L-fucose mutarotase	7.123
			L-fuculokinase (EC 2.7.1.51)	6.274
			L-fuculose phosphate aldolase (EC 4.1.2.17)	4.879
	D-gluconate and ketogluconates metabolism	NA	Hypothetical oxidoreductase YqhD (EC 1.1.-.-)	4.852
Cell wall and capsule	Major outer membrane proteins	NA	Attachment invasion locus protein precursor	5.199
	Capsular polysaccharides biosynthesis and assembly	NA	UDP-N-acetylglucosamine 4-epimerase (EC 5.1.3.7)	4.685
Fatty acids, lipids, and isoprenoids	Carnitine metabolism in microorganisms	NA	Crotonobetainyl-CoA dehydrogenase (EC 1.3.99.-)	7.587
Protein metabolism	Glycine reductase, sarcosine reductase and betaine reductase	NA	Glycine reductase component B gamma subunit (EC 1.21.4.2)	20.327
	Protein chaperones	NA	Chaperone protein HscB	4.736
Respiration	Na+ translocating decarboxylases and related biotin-dependent enzymes	NA	Methylmalonyl-CoA:Pyruvate transcarboxylase 12S subunit (EC 2.1.3.1)	5.247
RNA metabolism	Transcription initiation, bacterial sigma factors	NA	RNA polymerase principal sigma factor HrdD	5.355
	ATP-dependent RNA helicases, bacterial	NA	ATP-dependent RNA helicase YqfR	−2.919
Stress response	Periplasmic acid stress response in Enterobacteria	9.966	Chaperone HdeA	8.802
	Acid resistance mechanisms	6.554	Probable glutamate/gamma-aminobutyrate antiporter	9.128
			Glutamate decarboxylase (EC 4.1.1.15)	6.477
	Glutaredoxins	NA	Glutaredoxin 2	5.631
	Universal stress protein family	NA	Universal stress protein D	7.688
Sulfur metabolism	Galactosylceramide and sulfatide metabolism	NA	Alpha-galactosidase (EC 3.2.1.22)	3.542
Others	Conserved cluster in *Enterobacteriaceae* downstream from YqjA, a DedA family protein	NA	Inner membrane protein YqjK	5.302

*A positive number in the Log2 fold change means that the breast milk group has up-regulation on that function compared to the formula group; a negative number means down-regulation in the breast milk group. The “NA” in the Log2 fold change column means that this function has no significant difference between two groups (*p*_*adj*_ > 0.05).

## Discussion

Studies on preterm infant gut microbiome have been relatively few because of difficulty in obtaining samples. A few studies reported the feeding impacts on very preterm (Cong et al., 2016) and extremely preterm infants (Gewolb et al., 1999), but the moderate–late preterm cohort composes the majority of the preterm neonate population. Consequently, studies on the moderate–late preterm neonate gut microbiome focusing on nutritional effects have been generally lacking.

Regarding the alpha-diversity between different nutritional sources, there are no significant differences in alpha-diversity between MBM- and formula-fed preterm infants. The beta-diversity in the genus level, however, was found to be different whereas there was not a significant difference at the phylum level. Based on the 16S rRNA amplicon approach, at the phylum level, *Firmicutes* predominates, followed by *Proteobacteria* in both groups. At the genus level, samples of the MBM group were primarily dominated by *Veillonella* and *Escherichia/Shigella*, followed by *Staphylococcus*, *Enterococcus*, *Clostridium*, *Streptococcus*, and *Klebsiella*. In contrast, in the formula-fed group, *Streptococcus* and *Klebsiella* were the main colonizers, while *Enterococcus*, *Staphylococcus*, *Veillonella*, and *Clostridium* were the minor colonizers. In this study, using both balanced and unbalanced methods to analyze the differentially abundant taxa, we discovered that *Propionibacterium* is consistently more abundant in the MBM-fed group than in the formula-fed group. A previous study associated the presence of *Propionibacterium* in the guts of infants with the mother’s skin flora, which may be related to the colonization of *Propionibacterium* during the cesarean section process (Dominguez-Bello et al., 2010). However, after we analyzed the data based on other groupings, including the delivery mode, we did not find a significant difference in *Propionibacterium* besides the grouping of nutrition source. This suggests that the significant difference of *Propionibacterium* in this study is mainly associated with the nutrition source. *Propionibacterium* is usually considered a beneficial bacterium (Holzapfel et al., 2001; Kechagia et al., 2013), although not a dominator in the gut core microbiome. Moreover, our results from UPARSE machine learning also confirmed that *Propionibacterium* was the most predictive indicator for distinguishing between the MBM- and formula-fed preterm infants. In addition to *Propionibacterium*, Gneiss results also showed that MBM infants had a significantly greater population from *Streptococcus* and *Finegoldia* genera than those of the formula group, but less from family *Enterobacteriaceae* and genus *Enterococcus*, which are basically consistent with the metatranscriptomic results.

In this study, we found no difference in the relative abundance of *Bifidobacterium* and *Lactobacillus* between MBM- and formula-fed groups. *Bifidobacterium* and *Lactobacillus* are both considered to be beneficial and are usually dominant in full-term healthy infants (Stark and Lee, 1982; Benno et al., 1984; Balmer and Wharton, 1989; Bezirtzoglou et al., 2011). Similar to previous studies in preterm infants (Gewolb et al., 1999; Palmer et al., 2007; Cong et al., 2016; Wandro et al., 2018), we found generally low relative abundances of *Bifidobacterium* and *Lactobacillus* based on both 16S rRNA gene amplicon sequencing and metatranscriptomics approaches, except for one sample in each group. Although the DNA extraction methods and the selection of PCR primers were speculated to result in the artifact of the underrepresentation of *Bifidobacteria* (Gomez-Llorente et al., 2013; Walker et al., 2015), through the metatranscriptomic approach, which should have eliminated the bias, we found that *Bifidobacteria* indeed had a relatively low population. A possible explanation of this phenomenon of low relative abundance in *Bifidobacterium* is due to the gestation age (Butel et al., 2007; Arboleya et al., 2012; Chernikova et al., 2018). It is plausible that the moderate–late preterm infant intestine is not developmentally ready for this type of colonization. Alternatively, the extended hospital stay of preterm infants leads to dysbiosis due to a difference in environmental exposures from a home setting (Schwiertz et al., 2003; Magne et al., 2006; Roudière et al., 2009; Rougé et al., 2010; Jacquot et al., 2011), and antibiotic use intrapartum or direct use for neonates (Zimmermann and Curtis, 2019) may have contributed to the low abundances regardless of nutrition source.

The different levels of L-fucose metabolic genes in the two groups may be one of the reasons for the differences in bacterial composition. Some studies have shown that human milk oligosaccharides (HMO) are abundant in human breast milk but cannot be absorbed by infants. However, HMO serve as a metabolic substrate for specific bacteria in the infant gut (Engfer et al., 2000; Gnoth et al., 2000). L-fucose is one of the monomers of HMO and is very abundant in the human gut with several common human gut bacteria including *Bifidobacterium*, *E. coli*, and several species of *Bacteroides* known to utilize the L-fucose (Eagon, 1961; Pacheco et al., 2012; Bunesova et al., 2016; Westreich et al., 2019). Based on our results, we speculate that MBM infants had access to more L-fucose as an oligosaccharide than did formula infants, which could have selected for certain bacteria in the infant gut (Bode, 2009). The acidity of the gut resulting from nutritional sources may also play a role in affecting the neonates’ gut microbiome. One study found that the pH of fecal samples in MBM infants (5.49 ± 0.4) was significantly lower than that of formula-fed infants (6.91 ± 0.8) 14 days after birth (Balmer and Wharton, 1989), which could serve as one of the explanations for the increased acid resistance mechanism of the MBM group we observed. Notably, the glycine reductase component B gamma subunit increased by a 20-log_2_ fold change in the MBM group. Glycine serves as an electron acceptor and is reduced by glycine reductase produced by anaerobic bacteria (Andreesen, 1994). The colonization of obligate anaerobes like *Streptococcus anginosus*, *Clostridium butyricum*, and *Veillonella dispar* tends to have a fermentation-based metabolism (Brown et al., 2013), which supports the observation of dominant *Streptococcus*, *Clostridium*, and *Veillonella* in this study’s MBM group. Acid resistance gene expression was likely upregulated in the MBM group because *Streptococcus*, *Clostridium*, and *Veillonella* produced acid during respiration through fermentation. Whether the increased gene expression in acid resistance and glycine reductase was connected to HMO utilization still needs to be verified in future studies including which of the bacteria are more effective.

Appropriate amino acid content in formula milk could be very important, especially for preterm neonates (Jang et al., 2018). In this study, we found an upregulation of the pathway responsible for the degradation of two essential amino acids, methionine and valine, in formula milk-fed neonate guts. We therefore speculate that the amino acid content in nutritional sources may exert selection pressure on specific members of the microbiome. As the protein source of most infant formula milk is based on cow milk whey, other studies comparing the concentration of amino acids in plasma of infants also indicated that the infants fed with whey-predominant formula had increased levels of valine and methionine (Janas et al., 1985; Schanler and Garza, 1987). Hypermethioninemia is a metabolic disorder in which most patients do not exhibit symptoms. However, for the few patients that do exhibit symptoms, they show clinical neurological disease (Chou, 2000; Chamberlin et al., 2000) and mild hepatitis (Augoustides-Savvopoulou et al., 2003; Baric et al., 2004). A study showed that 9 out of 10 hypermethioninemia babies were related to the intake of formula milk with excess methionine (Mudd et al., 2003). Cerebral edema in two of these patients was observed by brain MRI, and they returned to normal after reducing the amount of ingested methionine. Hypermethioninemia is caused by the deficiency of glycine N-methyltransferase (GNMT) activity (Augoustides-Savvopoulou et al., 2003; Luka and Wagner, 2003; Luka et al., 2006) and/or mutations of the MAT1A gene (Chou, 2000; Chamberlin et al., 2000), which resulted in the plasma concentration of methionine reaching as high as 1870 μM (normal range 5–35 μM). Therefore, more studies are required to understand the effect of these two amino acids on the gut–brain axis with respect to neurological disease. Reducing excessive methionine and valine in formula may be needed to mitigate the occurrence of disease.

16S rRNA gene amplicon sequencing is commonly used to determine the gut microbiome, but limitations exist including uneven PCR amplification, short sequence lengths (Quince et al., 2009; Poretsky et al., 2014), and low-accuracy OTU mapping (Huse et al., 2010; Ranjan et al., 2018). In recent years, the rapid development of high-throughput sequencing technology and reduced costs has made metatranscriptome feasible in microbiome studies. In contrast to 16S rRNA gene amplicon sequencing, metatranscriptome directly compares the gene function expression and dynamic changes (Bashiardes et al., 2016; Shakya et al., 2019). In this study, we found most of the differences in bacterial composition between the MBM group and the formula-fed group to be consistent. However, there is a slight difference in the proportion of bacteria between the two methods. In the metatranscriptome results, the proportion of *Streptococcus* in the MBM group was more than it was in the formula group, which was the opposite according to the 16S syntax pie charts. The differential abundance analysis based on Gneiss for 16S sequencing data did agree with the metatranscriptome results that *Streptococcus* was more abundant in the MBM group than in the formula group. We also found that *Veillonella* and *Klebsiella* were lowly represented in metatranscriptome results, whereas *Bifidobacteria* and *Pantoea* were highly represented compared to the 16S rRNA gene amplicon sequencing results. In this study, we simultaneously extracted DNA and RNA for 16S rRNA gene amplicon sequencing and metatranscriptome analysis, using the same grinding step. This step ruled out that some bacteria known to be under-represented is caused by insufficient grinding using different kits during the nucleic acid extraction step. Therefore, we speculate that the differences observed between the two approaches are mainly due to the selective amplification of PCR, which needs to be verified by side-by-side comparisons of pre-defined composition of microbiomes in a future study.

The main limitation of this study was that the collection of fecal samples from preterm infants staying in a neonatal intensive care unit (NICU) was a major obstacle. The sampling scheme in this study managed to eliminate as many confounding factors as we can readily perceive. For example, we are aware that C-section versus vaginal delivery is a factor known to affect the microbiome in infants, so there was not a statistical difference between numbers of infants delivered by each method within our study groups. However, one of the unique characteristics of this study is that it included a relatively high number of Native American infants. To our knowledge, the microbiome of Native American infants has not been well evaluated within other studies. The infants enrolled in our study well-represent the overall demographics of NICUs in our region. We had not previously evaluated the breast-fed versus formula-fed rates between ethnical demographics in our NICU as breast-feeding is promoted to all populations regardless of demographics. Therefore, we did not anticipate a difference in race between our groups during recruitment. There may be population health implications in our findings which would be of interest to future studies. Also, in practicality, twins were not excluded from the study as twin sets are common in NICUs as they are more likely to be delivered preterm than singletons. Although exposures were similar, we did not note identical microbiomes for twin sets.

This study is clinically relevant to preterm infants during their stay at NICUs because certain diseases such as NEC tend to happen in NICUs with extreme- to very-preterm populations who have immature gastrointestinal tract. As the first comprehensive investigation to compare the effects of nutrition on early microbial colonization of preterm infant gut, this study focused on not only taxonomy but also function through 16S rRNA and metatranscriptome methods. We found that the relative abundance of *Propionibacterium*, *Streptococcus*, and *Finegoldia* and order *Clostridiales* in the MBM group was significantly higher than that of the formula group, and the relative abundance of family *Enterobacteriaceae*, genera *Enterococcus* and *Veillonella*, and class *Bacilli* in the formula group was significantly higher than that of the MBM group. Probiotics, *Bifidobacterium* and *Lactobacillus*, did not have a significant difference between the two groups. Through functional analysis, we have found that the bacterial genes related to glycine reductase, periplasmic acid stress response in *Enterobacteria*, acid resistance mechanisms, and L-fucose utilization functions in the MBM group were upregulated, while the methionine and valine degradation functions were downregulated compared to the formula group. This article has provided a deeper insight into the shaping of the gut microbiome in preterm infants by nutrition, which will be valuable to the improvement of formula milk for this population.

## Data Availability Statement

The datasets generated for this study can be found in the NCBI SRA PRJNA515307.

## Ethics Statement

The studies involving human participants were reviewed and approved by the Sanford Health. Written informed consent to participate in this study was provided by the participants’ legal guardian/next of kin.

## Author Contributions

ZW, AN, and S-YM analyzed the sequencing data. ZW constructed the metatranscriptome libraries. JW, XW, and S-YM conceived the study. ZW, S-YM, and RV helped to draft the manuscript. All authors read and approved the final manuscript.

## Conflict of Interest

The authors declare that the research was conducted in the absence of any commercial or financial relationships that could be construed as a potential conflict of interest.

## Footnotes

1 https://github.com/transcript/macaqueICD
