# Supplementary material for: Comparing Gut Microbiome in Mothers’ Own Breast Milk- and Formula-Fed Moderate-Late Preterm Infants
Source: Front Microbiol. 2020 May 26;11:891. doi: 10.3389/fmicb.2020.00891 (PMC7264382; doi:10.3389/fmicb.2020.00891)
Supplement: Supplementary file 1 [file Data_Sheet_1.pdf]

Supplementary:

Supplementary Table S1. Population demographics

	Breast milk	Formula	p value
Gestational age(weeks)	33.7 ± 1.26	34 ± 1.5	0.6546
Birth weight(grams)	2114 ± 415	2202 ± 494	0.6702
First sample age(days)	15 ± 6.4	15 ± 14.3	0.4114
Second sample age(days)	17 ± 5.9	17 ± 14.9	0.9951
Antibiotic exposure	60%	50%	
Vaginal delivery	30%	30%	
Gender(male)	30%	60%	
Twins	40%	60%	
Proton Pump Inhibitor	0%	0%	

Supplementary Table S2. Alpha diversities in different nutrition.

Index				p-value
berger_parker	Fomular	~	Breast milk	0.48
buzas_gibson	Fomular	~	Breast milk	0.684
chao1	Fomular	~	Breast milk	0.794
dominance	Fomular	<	Breast milk	0.142
equitability	Fomular	<	Breast milk	0.123
jost	Fomular	<	Breast milk	0.124
jost1	Fomular	<	Breast milk	0.141
reads	Fomular	<	Breast milk	0.142
richness	Fomular	~	Breast milk	0.483
richness2	Fomular	~	Breast milk	0.315
robbins	Fomular	<	Breast milk	0.164
simpson	Fomular	>	Breast milk	0.142
shannon_e	Fomular	<	Breast milk	0.142
shannon_2	Fomular	<	Breast milk	0.143
shannon_10	Fomular	<	Breast milk	0.143
flyvbjerg	Fomular	~	Breast milk	0.529
mirror	Fomular	<	Breast milk	0.19
mirrorns	Fomular	~	Breast milk	0.852
logfit	Fomular	>	Breast milk	0.137
logfitns	Fomular	~	Breast milk	0.222
logfitmu	Fomular	>	Breast milk	0.192
logfitmuns	Fomular	~	Breast milk	0.337

"~" and "=" = metric is approximately equal in both groups
">" and "<" = metric has weak significance (P < 0.2)
">>" = metric has high significant (P < 0.05)

Supplementary Table S3. Top 10 informative OTUs identified by random forest classifier[116] that can be used to distinguish MBM from formula-fed infants.

OTU number	Importance *	Frequency category**	Taxonomy predictions
Otu19	0.0137	Breast milk	d:Bacteria,p:Actinobacteria,c:Actinobacteria,o:Actinomycetales,f:Propionibacteriaceae,g:Propionibacterium
Otu34	0.0095	Formula	d:Archaea,p:Thaumarchaeota,o:Nitrososphaerales,f:Nitrososphaeraceae,g:Nitrososphaera
Otu282	0.00933	Formula	d:Others
Otu4	0.00887	Breast milk	d:Bacteria,p:Firmicutes,c:Bacilli,o:Bacillales,f:Staphylococcaceae,g:Staphylococcus
Otu26	0.00785	Formula	d:Bacteria,p:Actinobacteria,c:Actinobacteria,o:Actinomycetales,f:Micrococcaceae,g:Rothia
Otu77	0.00636	Formula	d:Archaea,p:Thaumarchaeota,o:Nitrososphaerales,f:Nitrososphaeraceae,g:Nitrososphaera
Otu329	0.00573	Formula	d:Bacteria,p:Actinobacteria,c:Actinobacteria
Otu69	0.00549	Formula	d:Bacteria,p:Acidobacteria,c:Acidobacteria_Gp4
Otu3	0.00517	Breast milk	d:Bacteria,p:Proteobacteria,c:Gammaproteobacteria,o:Enterobacteriales,f:Enterobacteriaceae,g:Escherichia/Shigella
Otu37	0.00507	Breast milk	d:Bacteria,p:Firmicutes,c:Bacilli,o:Lactobacillales,f:Streptococcaceae,g:Streptococcus

* The "Importance" value of a less than 0.05 indicates the significance of the particular otu in distinguishing between the two groups.

** Frequency category indicates the group showing the frequency of a particular otu.

Supplementary Table S4. The top 30 frequency distance rank of OTUs in nutrition.

Rank	Min Gini	Max AUC	AUC*	ScoreA	ScoreG	Med	Breast milk**	OTU Name	Taxa Name
**1**	0.2308	0.85	0.835	4	4	0	+	Otu19	**g:Propionibacterium**
**2**	0.32	0.8	0.76	0.5	0.5	1	-	Otu130	**c:Acidobacteria_Gp6**
**3**	0.3333	0.75	0.75	0.5	0.5	0	-	Otu282	**g:Oceanobacillus**
**4**	0.3333	0.75	0.675	2.30E+02	2.30E+02	0	+	Otu3	**g:Escherichia/Shigella**
**5**	0.3333	0.75	0.75	1	1	0	+	Otu63	**g:Veillonella**
**6**	0.3333	0.75	0.75	0.5	0.5	0	-	Otu571	**g:Subdivision3_genera _incertae_sedis**
**7**	0.3333	0.75	0.75	0.5	0.5	0	-	Otu49	**g:Enterococcus**
**8**	0.3333	0.75	0.775	1.5	1.5	0	-	Otu117	**g:Terrimonas**
**9**	0.3626	0.75	0.61	2.50E+02	2.50E+02	4.60E+02	+	Otu4	**g:Staphylococcus**
**10**	0.3626	0.75	0.74	1.5	1.5	0	-	Otu26	**g:Rothia**
**11**	0.3737	0.75	0.75	0.5	0.5	0	-	Otu150	**o:Actinomycetales**
**12**	0.375	0.7	0.7	4.5	4.5	0	-	Otu689	**g:Enterococcus**
**13**	0.375	0.7	0.72	1.5	1.5	0	-	Otu77	**g:Nitrososphaera**
**14**	0.375	0.7	0.7	0.5	0.5	0	-	Otu366	**g:Hyphomicrobium**
**15**	0.375	0.7	0.7	0.5	0.5	0	-	Otu329	**c:Actinobacteria**
**16**	0.375	0.7	0.7	0.5	0.5	0	-	Otu167	**o:Rhizobiales**
**17**	0.375	0.7	0.7	0.5	0.5	0	-	Otu248	**g:Gp4**
**18**	0.375	0.7	0.7	0.5	0.5	0	+	Otu1010	**g:Propionibacterium**
**19**	0.4048	0.7	0.675	0.5	0.5	0	-	Otu250	**g:Gp6**
**20**	0.4048	0.7	0.65	1.5	1.5	0	-	Otu90	**f:Geodermatophilaceae**
**21**	0.4048	0.7	0.695	6.5	5.5	5	-	Otu34	**g:Nitrososphaera**
**22**	0.4048	0.7	0.675	0.5	0.5	0	+	Otu14	**g:Finegoldia**
**23**	0.4118	0.65	0.65	0.5	0.5	0	-	Otu825	**g:Streptomyces**
**24**	0.4118	0.65	0.65	0.5	0.5	0	-	Otu238	**d:Bacteria**
**25**	0.4118	0.65	0.65	0.5	0.5	0	-	Otu281	**c:Actinobacteria**
**26**	0.4118	0.65	0.65	0.5	0.5	0	-	Otu359	**o:Actinomycetales**
**27**	0.4118	0.65	0.65	0.5	0.5	0	-	Otu392	**c:Betaproteobacteria**
**28**	0.4118	0.65	0.65	0.5	0.5	0	-	Otu259	**g:Gp6**
**29**	0.4118	0.65	0.65	0.5	0.5	0	-	Otu10	**g:Akkermansia**
**30**	0.4118	0.65	0.65	0.5	0.5	0	-	Otu23	**g:Dermabacter**

* AUC = Area under the ROC curve

** (+) or (-) indicates whether high or low abundance implies the positive category.

Supplementary Table S5. List of Gene Ontology Categories of two diet groups. Interpro2GO results of nine MBM infants and seven formula fed infants are showed. It is listed by decrease order.

MBM group	Formula fed group
GO:0003824 catalytic activity	GO:0003824 catalytic activity
GO:0008152 metabolic process	GO:0008152 metabolic process
GO:0003676 nucleic acid binding	Other
GO:0043167 ion binding	GO:0031224 intrinsic component of membrane
Unclassified	GO:0006810 transport
GO:0006810 transport	Unclassified
GO:0006259 DNA metabolic process	GO:0071840 cellular component organization or biogenesis
GO:0031224 intrinsic component of membrane	GO:0016070 RNA metabolic process
Other	GO:0003676 nucleic acid binding
GO:0006950 response to stress	GO:0000166 nucleotide binding
GO:0000166 nucleotide binding	GO:0043167 ion binding
GO:0071840 cellular component organization or biogenesis	GO:0005215 transporter activity
GO:0016070 RNA metabolic process	GO:0006259 DNA metabolic process
GO:0005215 transporter activity	GO:0003735 structural constituent of ribosome
GO:0005622 intracellular	GO:0005515 protein binding
GO:0016020 membrane	GO:0006950 response to stress
GO:0003735 structural constituent of ribosome	GO:0006457 protein folding
GO:0005840 ribosome	GO:0016020 membrane
GO:0005737 cytoplasm	GO:0005737 cytoplasm
GO:0098796 membrane protein complex	GO:0046906 tetrapyrrole binding
GO:1902494 catalytic complex	GO:0005622 intracellular
GO:0019222 regulation of metabolic process	GO:0005840 ribosome
GO:0050662 coenzyme binding	GO:0050662 coenzyme binding
GO:0005515 protein binding	GO:1902494 catalytic complex
GO:0030170 pyridoxal phosphate binding	GO:0098796 membrane protein complex
GO:0006457 protein folding	GO:0019222 regulation of metabolic process
GO:0003700 DNA-binding transcription factor activity	GO:0007165 signal transduction
GO:0042221 response to chemical	GO:0030170 pyridoxal phosphate binding
GO:0046906 tetrapyrrole binding	GO:0003700 DNA-binding transcription factor activity
GO:0051536 iron-sulfur cluster binding	GO:0019842 vitamin binding
GO:0016209 antioxidant activity	GO:0016032 viral process
GO:0019842 vitamin binding	GO:0009628 response to abiotic stimulus
GO:0009628 response to abiotic stimulus	GO:0016209 antioxidant activity
GO:0004601 peroxidase activity	GO:0042221 response to chemical
GO:0045454 cell redox homeostasis	GO:0045454 cell redox homeostasis
GO:0016226 iron-sulfur cluster assembly	GO:0004601 peroxidase activity
GO:0042597 periplasmic space	GO:0016226 iron-sulfur cluster assembly
GO:0005694 chromosome	GO:0051536 iron-sulfur cluster binding
GO:0007165 signal transduction	GO:0005694 chromosome
GO:0005618 cell wall	GO:0042597 periplasmic space
GO:0048500 signal recognition particle	GO:0038023 signaling receptor activity
GO:0009288 bacterial-type flagellum	GO:0071973 bacterial-type flagellum-dependent cell motility
GO:0009372 quorum sensing	GO:0048500 signal recognition particle
GO:0043934 sporulation	GO:0009372 quorum sensing
GO:0071973 bacterial-type flagellum-dependent cell motility	GO:0009405 pathogenesis
GO:0030246 carbohydrate binding	GO:0009346 citrate lyase complex
GO:0038023 signaling receptor activity	GO:0005576 extracellular region
GO:0009405 pathogenesis	GO:0043934 sporulation
GO:0005839 proteasome core complex	GO:0005634 nucleus
GO:0005576 extracellular region	GO:0009607 response to biotic stimulus
GO:0009579 thylakoid	GO:0030246 carbohydrate binding
GO:0009346 citrate lyase complex	GO:0005727 extrachromosomal circular DNA
GO:0007155 cell adhesion	GO:0005618 cell wall
GO:0006808 regulation of nitrogen utilization	GO:0005839 proteasome core complex
GO:0005727 extrachromosomal circular DNA	GO:0009288 bacterial-type flagellum
GO:0016032 viral process	GO:0007155 cell adhesion
GO:0005634 nucleus	GO:0009579 thylakoid
GO:0009607 response to biotic stimulus	GO:0006808 regulation of nitrogen utilization
GO:0005874 microtubule	GO:0005874 microtubule
	GO:0019012 virion

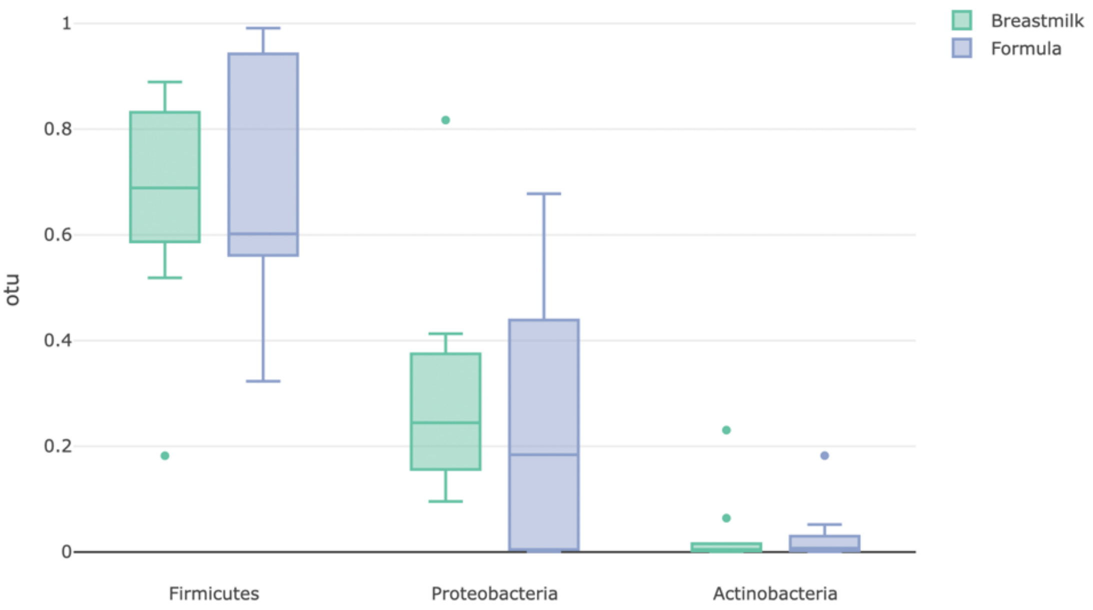

Supplementary Figure S1. Boxplot of the top three most abundant bacterial phyla in breast milk and formula fed preterm infants.

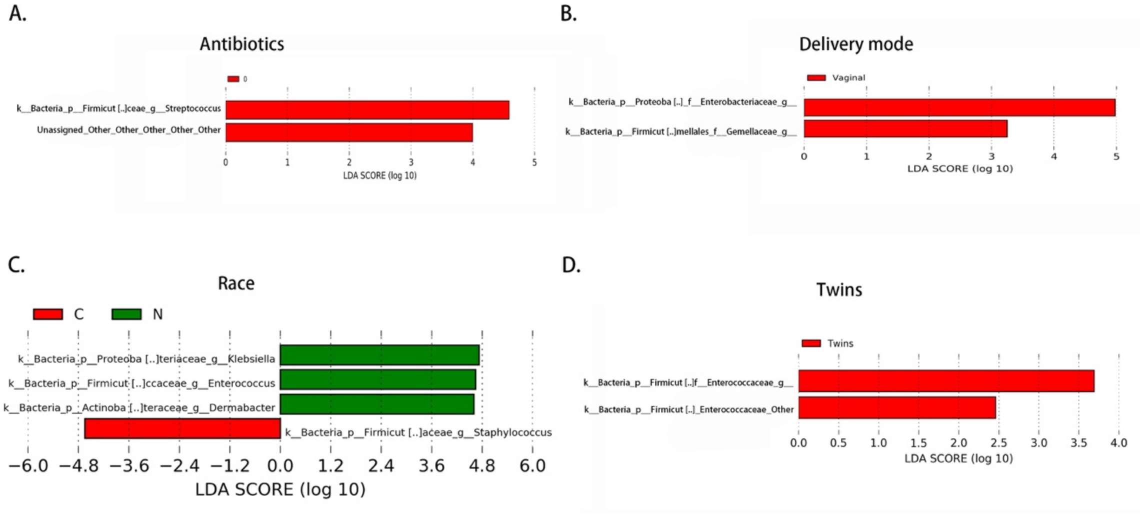

Supplementary Figure S2. Bar chart of the relative abundance differences between different factors. (A) Bar chart of the relative abundance differences between antibiotics and non-antibiotics (0). (B) Bar chart of the relative abundance differences between vaginal delivery and Caesarean section. (C) Bar chart of the relative abundance differences between Caucasian (C) and Native American (N). (D) Bar chart of the relative abundance differences between twins and non-twins.

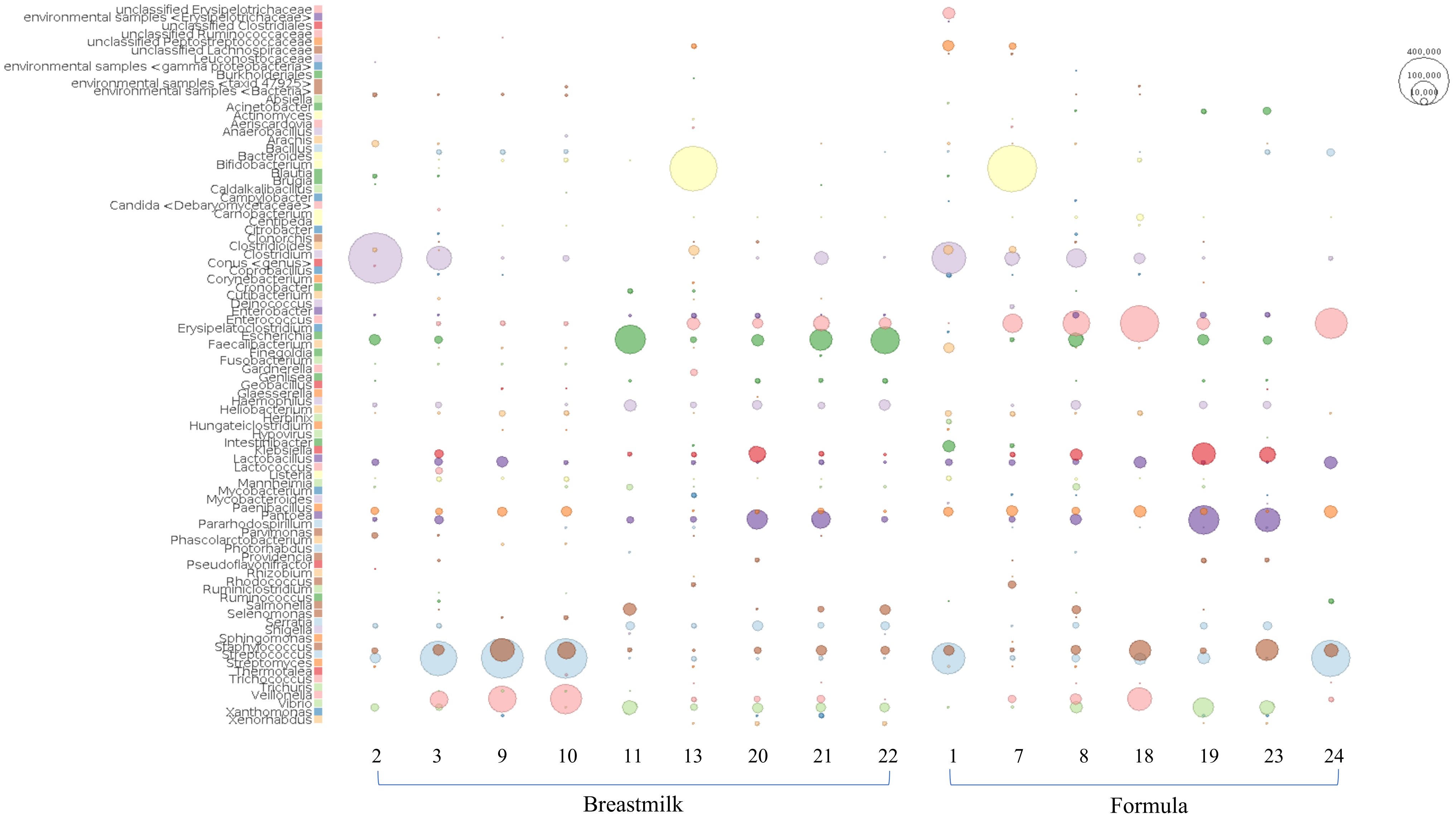

Supplementary Figure S3. Bubble charts of sixteen infants metatranscriptomic results at genus level

A)

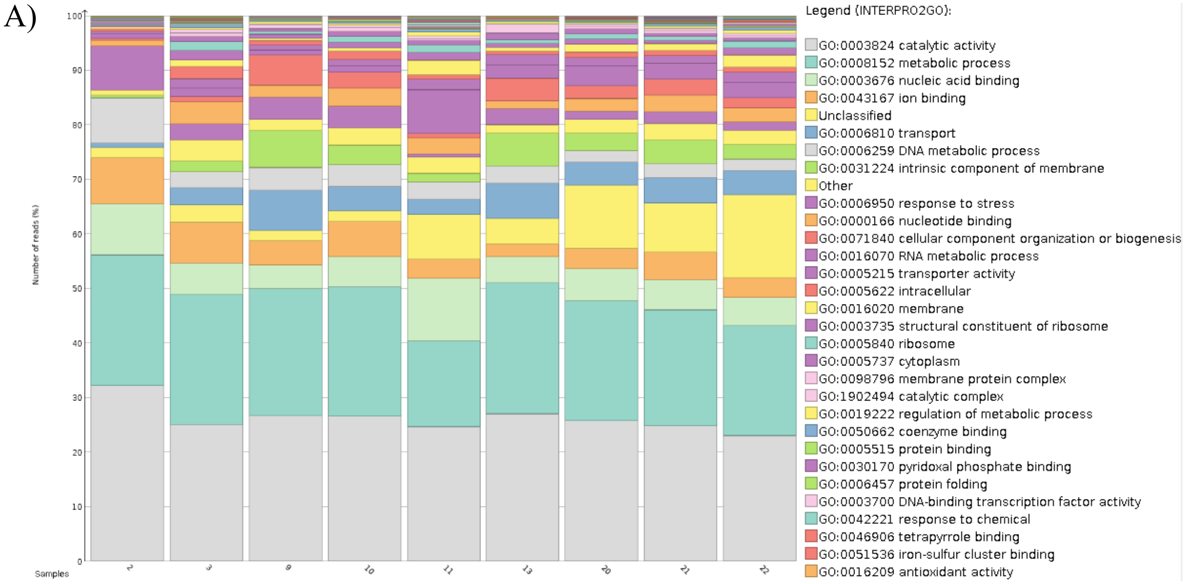

B)

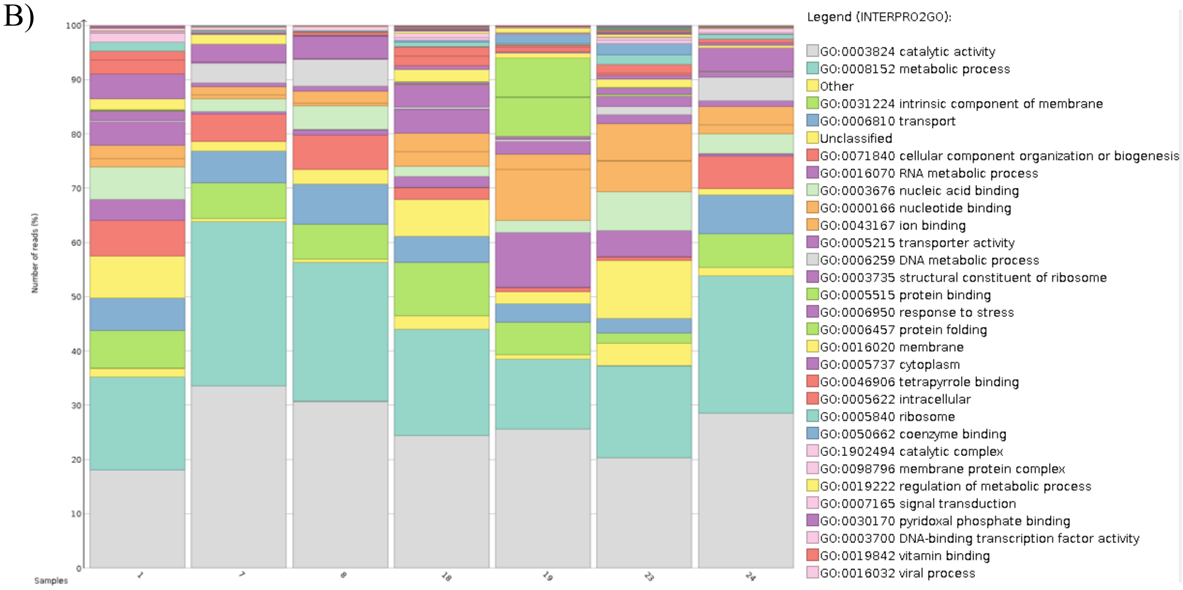

Supplementary Figure S4. Functional analysis (InterPro2GO) of sixteen infants. (A) Bar chart of InterPro2GO in MBM infants. (B) Bar chart of InterPro2GO in formula-fed infants (Partial legend was showed, see Supplementary Table 5 for complete list).

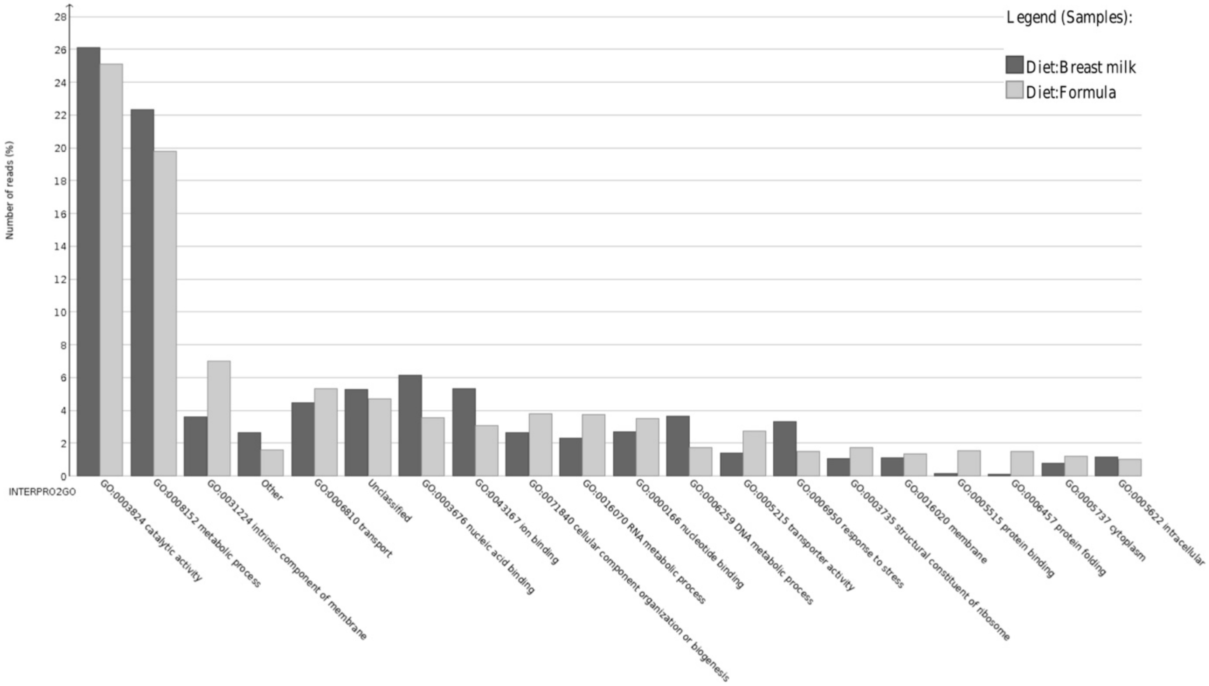

Supplementary Figure S5. Number of reads of Gene Ontology Categories compared between two diet groups. The summary of Interpro2GO results from nine MBM infants and seven formula fed infants. Partial categories are shown, listed by decrease order. Bar charts of gene ontology shown infants individually and the complete list of Gene Ontology Categories are in Supplemental Table 5.

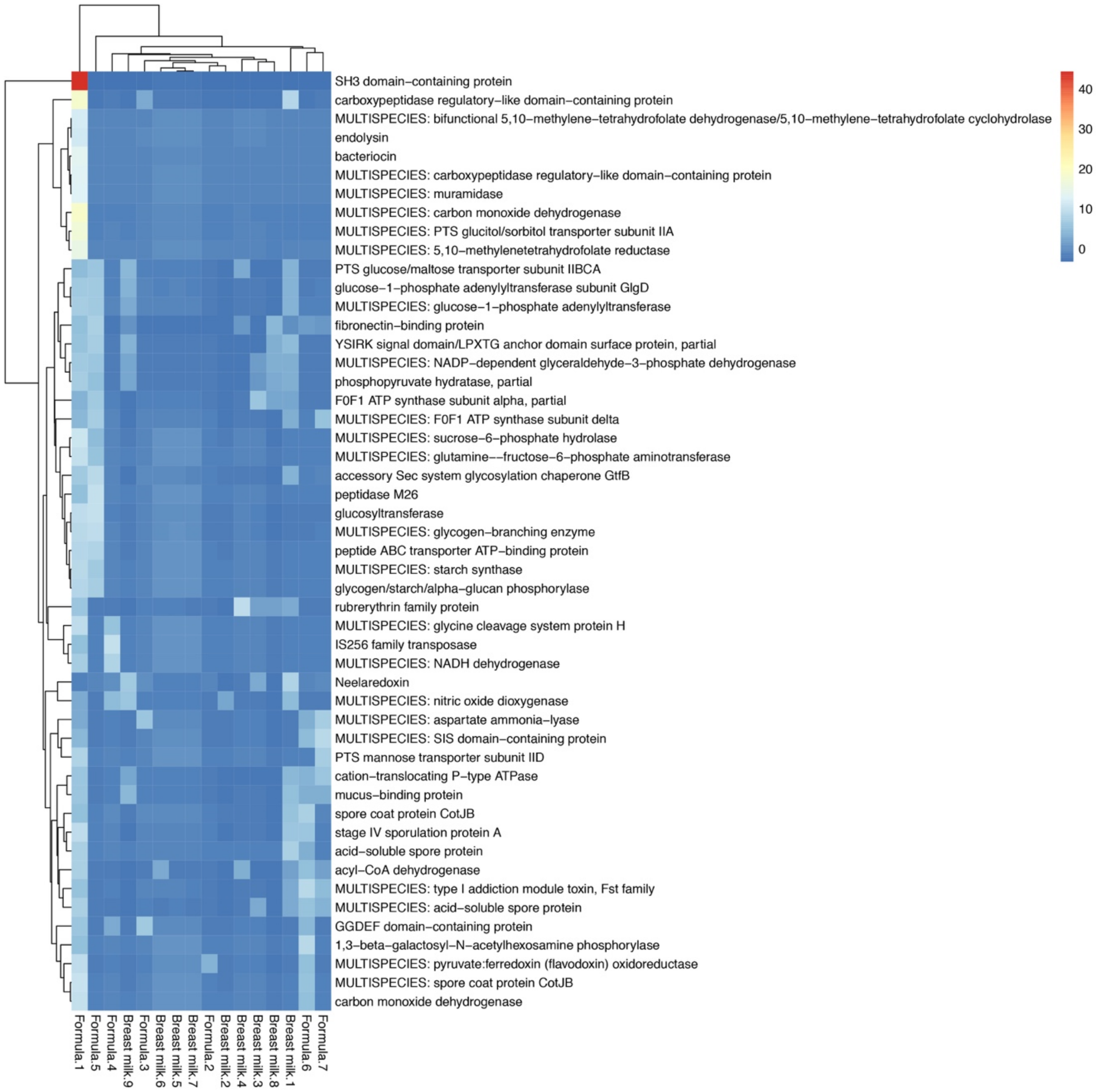

Supplementary Figure S6. Heatmap of function analysis against RefSeq database of sixteen infants.
